# Developing an Equitable Machine Learning–Based Music Intervention for Older Adults At Risk for Alzheimer Disease: Protocol for Algorithm Development and Validation

**DOI:** 10.2196/73711

**Published:** 2025-08-07

**Authors:** Chelsea S Brown, Luna Dziewietin, Virginia Partridge, Jennifer Rae Myers

**Affiliations:** 1 Health Sciences Integrated Program Feinberg School of Medicine Northwestern University Chicago, IL United States; 2 Musical Health Technologies Los Angeles, CA United States; 3 Center for Data Science and Artificial Intelligence University of Massachusetts Amherst Amherst, MA United States; 4 Department of Hearing and Speech Sciences University of Maryland College Park, MD United States

**Keywords:** Alzheimer disease, digital health, lifestyle, machine learning, music

## Abstract

**Background:**

Given the high prevalence and cost of Alzheimer disease (AD), it is crucial to develop equitable interventions to address lifestyle factors associated with AD incidence (eg, depression). While lifestyle interventions show promise for reducing cognitive decline, culturally sensitive interventions are needed to ensure acceptability and engagement. Given the increased risk for AD and health care barriers among rural-residing older adults, tailoring interventions to align with rural culture and distinct needs is important to improve accessibility and adherence.

**Objective:**

This protocol aims to develop an intelligent recommendation system capable of identifying the optimal therapeutic music components to elicit engagement and resonate with diverse rural-residing older adults at risk for AD. Aim 1 is to develop culturally inclusive user personas for rural-residing older adults to understand their goals and challenges for music-based digital health intervention. Aim 2 is to develop knowledge embedding–based machine learning (ML) models that use music metadata and survey response data to identify optimal therapeutic music components for enhancing engagement and emotional resonance for depression among rural-residing older adults at risk for AD. Aim 3 is to assess acceptability for personalized therapeutic music sessions and ML-based music recommendations with a separate sample.

**Methods:**

Participants (N=1200) will be aged 55 years or older and residing in the United States. In phase 1, participants (n=1000) will receive 5 randomized songs and complete a survey to understand the sentiment, cultural relevance, and perceived benefit for each song. Brief, researcher-created Likert surveys will be used. In phase 2, survey data will be used to develop ML algorithms in collaboration with the University of Massachusetts Amherst Center for Data Science and Artificial Intelligence. These ML models will be integrated into the digital music intervention and tested with a separate sample of 200 participants. Similar to phase 1, participants will be provided with sets of songs generated by the recommendation system based on the target goal (ie, to reduce depression). The recommendation accuracy of the ML algorithm will be assessed using multiple performance metrics, including root-mean-square error and normalized discounted cumulative gain as well as the mean acceptability score with a goal of 85% user acceptability.

**Results:**

Participant recruitment is complete for phases 1 and 2 as of June 2025. Data analysis for the results of aims 1, 2, and 3 are underway and results are expected to be published in the fall of 2025.

**Conclusions:**

This protocol seeks to use ML to improve the equitability and accessibility of a digital lifestyle intervention for AD.

**International Registered Report Identifier (IRRID):**

DERR1-10.2196/73711

## Introduction

The prevalence of Alzheimer disease (AD) is expected to increase from 55 million cases worldwide in 2021 to an estimated 78 million cases by 2030 [1]. In 2024, the estimated health care cost of AD in the United States was $360 billion [2]. By 2050, it is estimated that the total annual health care cost will be $140,012 per US individual with AD, adding up to a total annual cost of $1.5 trillion [3]. Given the rising societal impact of AD, there is an urgent need for more preventative interventions.

One such preventative intervention is a lifestyle-based approach focusing on addressing modifiable risk factors of AD, such as smoking, diet, and depression [4]. Research investigating lifestyle interventions has yielded promising results [5,6]. The Finnish Geriatric Intervention Study to Prevent Cognitive Impairment and Disability randomized controlled trial examined a 2-year multidomain intervention for adults aged 60 years or older aimed at nutrition, physical activity, cognitive training, social support, and vascular risk factors [5]. The trial intervention showed beneficial changes in cognition and risk of cognitive decline and demonstrates the promise of lifestyle interventions for reducing the risk of overall cognitive decline (control group odds ratio=1.31; intervention group odds ratio=1) [5]. Research has also shown that treating depression can significantly reduce the risk of dementia [7]. Lifestyle-based approaches hold promise for preventing AD when designed to be accessible, especially for those at high risk of developing AD.

Older adults in rural communities face unique health challenges that place them at an even greater risk for AD. Social determinants of health, such as health care access and social engagement, coupled with psychosocial barriers (eg, stigma and medical mistrust) often exacerbate their risk [8,9]. These individuals face greater barriers to accessing lifestyle interventions, including limited health care resources, unreliable transportation, and reduced access to psychosocial support [10]. However, the majority of rural residents have access to broadband internet services [11], meaning digital lifestyle interventions may offer a more feasible, accessible, and cost-effective way to mitigate AD risk compared to traditional methods [12].

Although emerging research highlights the promising utility of lifestyle interventions, there remains a critical need for culturally sensitive approaches. Rural communities are often underrepresented in the development of digital cognitive and mental health tools, despite being disproportionately affected by cognitive and mental risk factors [13]. Research shows that interventions designed to be culturally sensitive can result in higher adherence and engagement with the intervention [14,15]. Additionally, intentional and meaningful inclusion of rural perspectives during the development of digital health interventions ensures individuals from rural, underrepresented communities actively participate in shaping their cognitive health landscape. Given the potential benefits of lifestyle interventions, culturally engaging and accessible interventions are needed to ensure utility and adherence.

**Figure 1:**
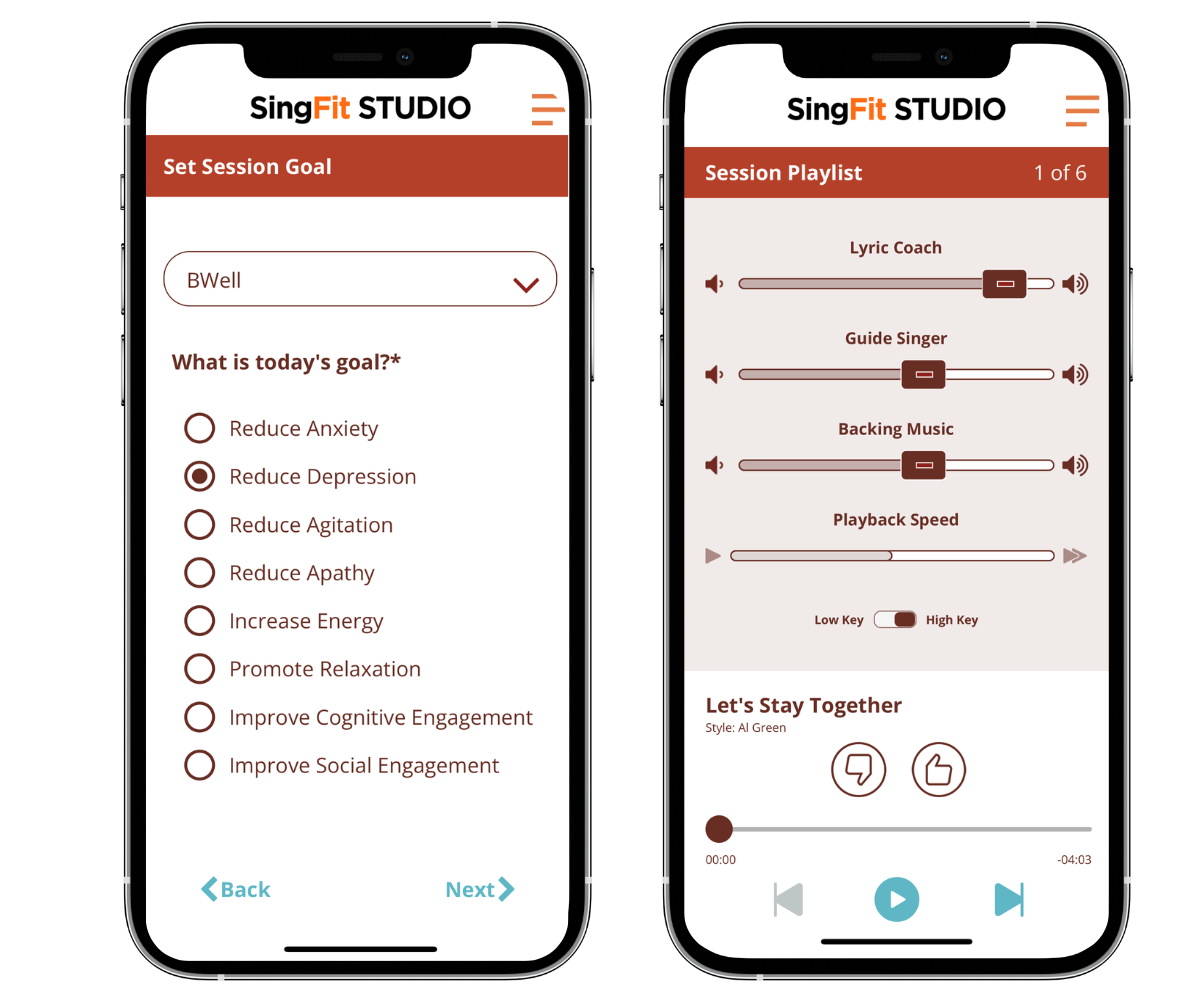
The SingFit mobile health app.

One digital music intervention, SingFit, was developed to improve well-being and mood symptoms for both those at risk for and those living with AD (Figure 1). The use of singing to manage symptoms of depression is based on research showing that music interventions and music learning are associated with improvements in cognitive reserve, well-being, and mood symptoms related to depression [16-19]. A network meta-analysis found evidence supporting the use of music therapy or music listening ≥60 minutes a week for improving depression in persons aged 60 years and older [20]. Furthermore, research has shown that music-based interventions reduce anxiety, agitation, and other neuropsychiatric symptoms associated with AD [21-23]. SingFit encourages active engagement in music-making by providing an auditory lyric prompter before each sung phrase. It uses music associated with each user’s reminiscence bump, which is the tendency for older adults to recall more personal memories associated with adolescence and young adulthood [24,25]. The app follows music therapy guidelines and clinical practice, where familiar, preferred music is often used to promote relaxation and positive affect [26]. This music is specifically chosen to elicit engagement, long term memory, and narrative speech. This intervention is further described in Brown and Myers [27].

Due to the need for accessible and culturally relevant lifestyle interventions aimed at reducing AD risk, this protocol seeks to improve the cultural responsiveness of an existing digital music-based intervention, SingFit, aimed at improving depressed mood for individuals at risk for or experiencing AD. Much like digital health tools, cultural considerations are important for music-based interventions. Older adults from rural communities tend to prefer traditional genres of music such as country, folk, and gospel; are less likely to embrace newer music trends; and are often influenced by their family’s and community’s musical preferences [28,29]. The purpose of this study is to develop a machine learning (ML) recommendation system driven by the attitudes and cultural preferences of rural-residing adults. Since acceptability and engagement are important to the success of any health intervention, cultural considerations are at the forefront of this intervention design [30]. This is reflected by the study’s aims, which are as follows:

1. Develop culturally inclusive user personas for rural-residing older adults to understand their goals and challenges for music-based digital health intervention.
2. Develop a knowledge embedding (KE)–based ML model that uses user data to identify optimal therapeutic music components for enhancing engagement and emotional resonance among rural-residing older adults at risk for AD.
3. Assess the acceptability of personalized therapeutic music sessions and ML-based music recommendations in a digital music intervention.

## Methods

### Participants

This survey study will lay the foundation for developing ML algorithms for an accessible music intervention for rural-residing older adults who are at risk for AD. Twelve hundred participants will be enrolled in the study. Eligibility for this study includes participants aged 55 years or older who have possession of a smartphone and the ability to read and understand English. Participants with self-reported presence of a diagnosed psychiatric or neurological condition, including AD, will be excluded. Recruitment was facilitated using a crowdsourcing research platform, Prolific.

### Ethical Considerations

The Salus Institutional Review Board determined that the present study was exempt in accord with 45 CFR 46.104(d)(2i, 2ii, 3A & 3B) on June 17, 2024. The exemptions fall under benign behavioral interventions or educational surveys where disclosure of responses would not result in risk of harm or civil liability, and where the information being recorded is anonymous or cannot be readily ascertained or linked to participants. Broad informed consent was obtained via an introductory paragraph on the survey, where participants were directed to continue with the survey to indicate acceptance. Participants were provided information on opting out of the study by emailing the principal investigator. Obtaining a signed informed consent would identify participants’ names, thus increasing the risk of linking information back to individuals. All participant data collected through Prolific was anonymized by the platform’s design. Prolific assigns participants a unique alphanumeric ID that is not linked to any personally identifying information. Researchers do not have access to names, email addresses, IP addresses, or any direct identifiers. Participants are paid $8 total for their participation in this study to complete the 45-minute survey. The CHERRIES (Checklist for Reporting Results of Internet E-Surveys) guidelines were used to guide the reporting of this protocol [31].

### Phase 1: Data Collection and Preprocessing

In phase 1, web-based survey data will be collected from up to 1000 older adults. Participants will be instructed to sing along to a playlist designed to reduce mood symptoms associated with depression. This goal was selected because depression is known to significantly increase the likelihood of AD and accelerate cognitive decline [32]. Participants will receive 5 songs randomly chosen from a set of songs based on age and musical arousal—2 factors known to be associated with mood and relevance to older adults. Songs will be selected based on participants’ “reminiscence bump” [24] and will use the iso principle to modulate from low arousal to moderate arousal [33,34]. In past studies of the musical “reminiscence bump,” the strongest associations and familiarity with music was found to be between the ages of 15-24 years [25,35]. SingFit’s music selections are chosen from participants’ adolescence and young adulthood to ensure familiarity and narrative memory associations with the music. The iso principle gradually shifts the music from matching the participant’s emotional state (eg, sad) to their desired state (eg, calm). In phase 1, songs with high arousal were not included in order to modulate arousal and affect gradually for a depression playlist in keeping with the iso principle. Surveys will use 5-point Likert scales to measure song familiarity, perceived benefit, sentiment, relevance, and preference for each song, which will be analyzed in conjunction with music metadata (described below) to better understand musical factors associated with mood symptoms of depression. Participants will receive an open survey link to the Qualtrics survey and either listen or sing along to each song while completing survey questions for each song (ie, song preference or sentiment).

For the development of user personas and the ML model (aims 1 and 2), the planned sample size of 1000 participants was determined following deliberation with the study’s data consultants to balance feasibility and analytical utility within the scope of this pilot project. This sample was identified as the minimum viable cohort necessary to support adequate model training, validation, and initial testing of the recommender system.

### Measures

Measures for this study are brief researcher-created surveys to measure song preference, expectation, and helpfulness for each song (see Multimedia Appendix 1). These measures were chosen to understand user preference in order to build a KE ML model and user personas for aims 1 and 2. These same measures will again be collected in a smaller sample for aim 3 to assess the acceptability of the ML-based music recommendations. Web-based surveys were developed by JRM and tested for usability and technical functionality by JRM and CSB prior to participant recruitment. Participants will be asked to answer each question for each song. Questions are 5-point Likert scales to measure song preference (strongly dislike to strongly like), sentiment (very sad to very happy), expectation for hearing that particular song for the goal of reducing depression (strongly disagree to strongly agree), and helpfulness (eg, “Would this song be helpful in making you feel better if you were very sad?”; strongly disagree to strongly agree). Participants will also be asked to assess their mood (eg, “How depressed are you?”) and engagement in daily activities prior to and directly following their survey on an 11-point Likert scale (0-10). Participant demographics will also be collected excluding directly identifying information that would link survey responses to participants.

Zip codes will be collected to verify geographical residence (rural, urban, or suburban) in order to build specific user personas tailored to each group (aim 1). After verification, only the first 3 digits of the zip code will be stored for each participant, and the digits will be changed to 000 in instances for which the zip codes contain less than 20,000 people.

Bot detection for survey entries based on reCAPTCHA was used, and any duplicate survey entries based on Prolific IDs were removed. Prolific uses participants’ email address and phone number to verify participants and IP address detection to ensure participants do not participate in studies more than once. There were an average number of 6.5 items per page with 8 total pages. All questions required an answer in order to submit the survey. Demographic questions included a “none of the above” or “prefer not to say” option, while music preference questions required a response on a 5-point Likert scale.

### Music Metadata

Each song in the SingFit catalog has been tagged with metadata for dozens of different parameters. Musical metadata include linguistic syntax and semantics (assessed with text analysis software), tempo and key (assessed using song analyzer software), genre, popularity, arousal, and others. The full list of metadata is proprietary to Musical Health Technologies but encompasses a wide range of cognitive and affective factors associated with known therapeutic benefits. These metadata will be used to understand the musical factors associated with user preference and clinical factors related to improvement in depressed mood. Music metadata is used in SingFit’s current song recommendation algorithms and will be used in knowledge graphs for comparing various KE model architectures during phase 1.

### Phase 2: Machine Learning Model Implementation and Evaluation

Music recommendation is an active area of research within the information retrieval community, with researchers facing unique challenges, such as data sparsity, complexity in evaluation, and personalization for cultural and situational awareness. The cold start problem, a lack of historical information for making decisions about new users or items, is a common issue across many recommendation systems that persists in the music domain [36,37]. Recent work has found promising results by using neural network models and incorporating metadata features such as song artist or genre with user interaction data to form a collaborative knowledge graph [37,38]. In particular, users, songs, and metadata categories are treated as nodes in a graph. Connecting edges represent semantic meaning, like a song belonging to a genre or a user favoriting a song. Neural network–based approaches like DeepWalk can be used for learning latent representations of the graph’s nodes in vector space [37,39]. At recommendation time, a song is compared to others in vector space, returning the most similar songs to the end user. This approach will allow the system to make recommendations based on the preferences of similar users in conjunction with shared music metadata and clinical features to maximize the relevance of recommendations.

The modeling architecture will largely follow Bertram et al [37] for creating knowledge graph embeddings of songs and relevant metadata features of both users and songs. Authors LD and VP will conduct explorations to vary the metadata features included in the knowledge graph and tune hyperparameters of the DeepWalk model. This will allow for thorough exploration of feature selection and model architecture choices, with the best performing modeling pipeline deployed for user testing. Models and data processing will be implemented in Python (Python Software Foundation) with widely used ML libraries, *scikit-learn* and *pytorch*, and support for graph structures with *pytorch-geometric* and *networkx*. The algorithms will be developed in collaboration with the data core at the University of Massachusetts Center for Data Science and the Massachusetts AI and Technology Center.

The data will be split into training, validation, and testing sets by user using 5-fold cross-validation. This will balance training and evaluation variances and allow for more efficient use of the relatively sparse data compared to using a single data split for training, validation, and testing sets. Splitting data at the user-level will simulate the cold start problem in our experiments. The model with the best average performance across the 3 test sets will be retrained on the entire dataset, a standard practice in ML. Baseline performance will be measured using a purely random recommendation system and a content-based learning-to-rank approach that relies solely on metadata features. These baseline benchmarks will help gauge the added benefit of more complex neural network recommendation algorithms.

Because clinical features may be in tension with musical preferences or musical features, multiple metrics are needed in order to understand the trade-off being made by the recommendation algorithm. The metrics that will be used to evaluate performance are root mean square error, normalized discounted cumulative gain, and a user-reported satisfaction score [36]. Root mean square error will be used to evaluate the error between the recommendation system’s estimated satisfaction score and the user’s true satisfaction score to ensure that it can accurately predict the user’s label of the song. Normalized discounted cumulative gain, a ranking quality metric, will be used on the songs recommended by the system to ensure that the test data songs are being recommended in an order equivalent to their user-preferred ranking. As part of further testing, a sample of users will be given a set of playlists recommended by the system and asked to answer the survey questions from phase 1. By comparing the satisfaction scores across phases, a comparison can be made as to the direct effects on the listener of the songs recommended.

For Aim 3, the goal is to develop a ML model with ≥80% accuracy in creating individualized therapeutic music playlists and achieve a user acceptability score of 85% or higher among rural-residing older adult users. Using the same survey questions from phase 1 (see Multimedia Appendix 1), the user acceptability score will be based on the average ratings for questions regarding the overall preference, relevance, and perceived benefit of the playlists. An a priori power analysis was conducted using G*Power version 3.1 [40] to determine the required sample size for a 1-sample *t* test for aim 3. The goal of the analysis was to detect a mean acceptability score significantly greater than 4.25 (indicating 85% acceptability), with a margin of error of 0.2 and an estimated SD of 1.2. Assuming a medium effect size (d=0.30), an α level of .05, and a desired power of 0.80, the analysis revealed that a minimum of 139 participants would be needed to ensure adequate statistical power.

## Results

**Figure 2:**
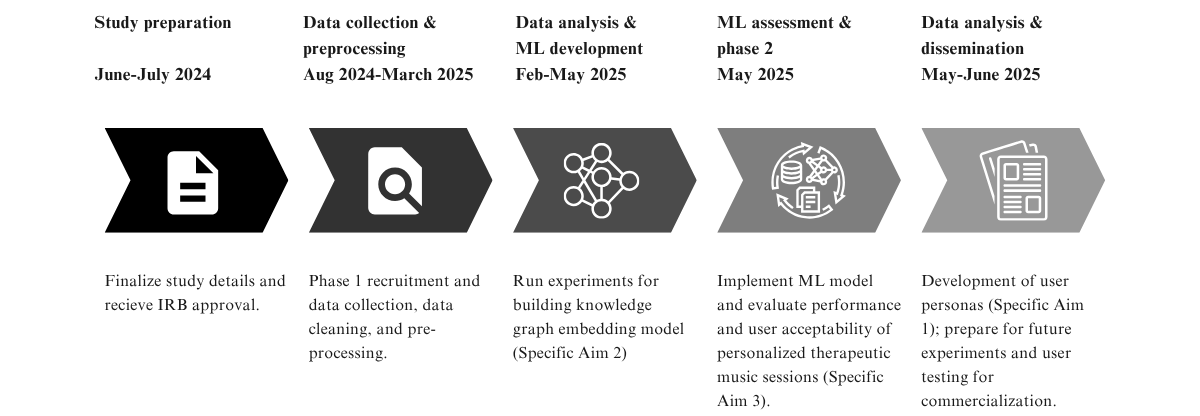
Study timeline. IRB: institutional review board; ML: machine learning.

Participant recruitment for the final protocol was initiated on December 31, 2024. In June of 2025, participants had completed the phase 1 and 2 web-based survey studies (Figure 2). Data preprocessing and analysis is underway to prepare publication of the results.

Due to challenges with participant recruitment and high attrition related to the initial study protocol, the study team made 2 changes to the initial study design. All modifications were administratively reviewed by the external institutional review board and subsequently implemented in compliance with ethical guidelines. First, the initial eligibility criteria for participants included 2 or more risk factors for AD based on the Australian National University-Alzheimer’s Disease Risk Index (eg, education level). An amendment to the original study protocol was made to the “at least 2 risk factors” eligibility requirement. Additionally, due to the high attrition noted, the survey removed the need to download the SingFit app and moved all songs (.mp3 files) to Qualtrics. Multiple recruitment methods were planned, including word-of-mouth, crowdsourcing platforms (eg, Prolific), social media, and web-based caregiving organizations. However, once the above changes were made, recruitment was accomplished rapidly on a crowdsourcing platform (Prolific).

## Discussion

### Principal Results

To our knowledge, this is the first digital singing intervention aimed at lifestyle factors associated with AD, such as depression, for those aged 55 years or older. It is important to improve intervention fit and cultural acceptability to ensure accessibility and usability for rural-residing older adults who may benefit the most from this type of intervention. While other music interventions are available, none are specifically aimed at managing symptoms of depression and cognitive reserve in cognitively healthy adults. Using ML to improve the existing intervention algorithms should improve music recommendations and engagement in end users. ML models should also allow the research team to better understand musical factors associated with sadness and depression symptoms, furthering knowledge on the most appropriate music interventions for mood symptoms in rural-residing older adults.

### Limitations

A significant limitation of this work is that the music files were moved from the app and hosted in Qualtrics due to issues with recruitment and retention in phase 1. Participants were not exposed to the app user interface during the music preference surveys in phases 1 and 2, which may have altered the experience of the music intervention. However, only elements present in the app (ie, length of song play, liking songs, and music metadata) were used for building the ML models. Second, participants with cognitive impairment or AD were excluded. This may limit the generalization of the recommendation system and findings to cognitively health older adults. Lastly, the sample size of this pilot project was constrained by the 1-year funding period. Although 1000 participants represents a meaningful sample for a pilot study, it is relatively small for training KE models and may impact the model’s predictive performance for music recommendations. Future phases of this project will include additional training and refinement of the model using larger datasets to enhance the model’s performance and overall generalizability.

### Comparison With Prior Work

While music interventions are not the first line of treatment for depression, they may provide an engaging, less invasive, ancillary treatment option for disengaged populations, such as rural-residing older adults or those with cognitive decline. When looking at comparative efficacy of depression treatments among individuals with dementia, cognitive stimulation (ie, reminiscence or art therapy) combined with exercise and social interaction or a cholinesterase inhibitor showed higher efficacy than some pharmacotherapies [41]. This demonstrates that psychosocial interventions such as music therapy or singing interventions that target multiple domains through cognitive stimulation, movement, and social interaction may provide valuable support for depression in those with AD. Music interventions provide a treatment approach for those with concerns of polypharmacy or those who may be unable to engage in psychotherapy (ie, those with AD).

A network meta-analysis showed moderate to high certainty evidence for active music interventions (ie, singing or playing instruments) at a dosage of ≥60 minutes per week in improving depression in adults aged 60 years or older [20]. An ongoing longitudinal study aimed at depression symptoms will improve our understanding of music interventions on both depression and neuroplasticity once the results are published [42]. This 3-group randomized controlled trial protocol compares 12 months of singing lessons versus physical activity or control (no intervention) in those with mild dementia or mild cognitive impairment [42]. Their primary hypothesis is that weekly singing can improve brain plasticity (brain age gap estimation measured by functional magnetic resonance imaging ) and depression (assessed using the geriatric depression scale) and secondarily improve cognition and activities of daily living. This study is still in progress, but should improve our understanding of the associations between music and physical activity interventions on decelerating or preventing AD.

Digital interventions can be valuable for improving accessibility to rural populations. A pilot project of a web-based music therapy program provided 3 sessions for tailored recommendations and music activities for persons with AD and their caregivers [43]. The intervention component focused on mood symptoms was to recommend singing between caregiver and the person with AD. Due to the small sample (n=13 caregiver and person with AD dyads), this study was not powered to show statistical significance, but it did demonstrate trends for ameliorating agitation—the mean neuropsychiatric inventory score for the agitation item preintervention was 1.0, while the postintervention score was 0.69—and improved the caregiving relationship, with 84.7% of participants reporting that music therapy improved communication between themselves and the person with AD. While web-based sessions improve the reach of these types of services for rural populations, this intervention lacks widespread scalability. To our knowledge, no other existing digital singing interventions have been aimed at AD risk factors.

### Conclusions

There is an urgent need to offer culturally sensitive digital lifestyle interventions for underrepresented populations at risk for AD to optimize long-term efficacy. Results from the protocol will contribute to the advancement of such interventions for older adults in rural communities by addressing the critical culture gap observed in digital health. Through the development of a KE-based ML recommendation system, this protocol seeks to improve the equitability and accessibility of a digital lifestyle intervention focused on reducing depression as a means of AD prevention.

## Appendix

Multimedia Appendix 1 Music preference survey.

## Abbreviations

AD: Alzheimer disease
CHERRIES: Checklist for Reporting Results of Internet E-Surveys
KE: knowledge embedding
ML: machine learning

## Data Availability

The data from this study are available upon reasonable request. Certain parts of the data, including music metadata, are proprietary to SingFit and cannot be hosted on a publicly available repository.
