# Supplementary material for: Developing an Equitable Machine Learning–Based Music Intervention for Older Adults At Risk for Alzheimer Disease: Protocol for Algorithm Development and Validation
Source: JMIR Res Protoc. 2025 Aug 7;14:e73711. doi: 10.2196/73711 (PMC12371280; doi:10.2196/73711)
Supplement: Multimedia Appendix 1 [file resprot_v14i1e73711_app1.docx]

**Appendix 1**

**Music Preference Survey**

**Song Title: ________**

**Please indicate your familiarity with this song.**

Not familiar

Moderately Familiar

Very Familiar

**Please Indicate your preference for this song.**

Strongly Dislike – Strongly Like

**Please rate the overall mood of the song.**

Very Sad

Sad

Neutral

Happy

Very Happy

**This song would help me feel better if I was very sad.**

Strongly Disagree – Strongly Agree

**I would expect the app to recommend this song for reducing depression.**

Strongly Disagree – Strongly Agree
